# Water and Meadow Views Both Afford Perceived but Not Performance-Based Attention Restoration: Results From Two Experimental Studies

**DOI:** 10.3389/fpsyg.2022.809629

**Published:** 2022-04-25

**Authors:** Katherine A. Johnson, Annabelle Pontvianne, Vi Ly, Rui Jin, Jonathan Haris Januar, Keitaro Machida, Leisa D. Sargent, Kate E. Lee, Nicholas S. G. Williams, Kathryn J. H. Williams

**Affiliations:** ^1^Melbourne School of Psychological Sciences, The University of Melbourne, Parkville, VIC, Australia; ^2^UNSW Business School, University of New South Wales, Sydney, NSW, Australia; ^3^School of Ecosystem and Forest Sciences, The University of Melbourne, Parkville, VIC, Australia

**Keywords:** sustained attention, attention restoration theory, pupillometry, connectedness to nature, SART, waterscape, meadow, alertness

## Abstract

Attention Restoration Theory proposes that exposure to natural environments helps to restore attention. For sustained attention—the ongoing application of focus to a task, the effect appears to be modest, and the underlying mechanisms of attention restoration remain unclear. Exposure to nature may improve attention performance through many means: modulation of alertness and one’s connection to nature were investigated here, in two separate studies. In both studies, participants performed the Sustained Attention to Response Task (SART) before and immediately after viewing a meadow, ocean, or urban image for 40 s, and then completed the Perceived Restorativeness Scale. In Study 1 (*n* = 68), an eye-tracker recorded the participants’ tonic pupil diameter during the SARTs, providing a measure of alertness. In Study 2 (*n* = 186), the effects of connectedness to nature on SART performance and perceived restoration were studied. In both studies, the image viewed was not associated with participants’ sustained attention performance; both nature images were perceived as equally restorative, and more restorative than the urban image. The image viewed was not associated with changes in alertness. Connectedness to nature was not associated with sustained attention performance, but it did moderate the relation between viewing the natural images and perceived restorativeness; participants reporting a higher connection to nature also reported feeling more restored after viewing the nature, but not the urban, images. Dissociation was found between the physiological and behavioral measures and the perceived restorativeness of the images. The results suggest that restoration associated with nature exposure is not associated with modulation of alertness but is associated with connectedness with nature.

## Introduction

Sustained attention is the skill of maintaining concentration and inhibiting distractions (Robertson et al., 1997), which is difficult to maintain and fluctuate over time (Williams et al., 2018). This important form of attention underpins more complex cognitive functions (Anderson and Doyle, 2004), and everyday tasks like driving (Robertson, 2003) and learning (Shannon et al., 2021), making it a good candidate for nature interventions. Attention Restoration Theory (ART) proposes that exposure to particular environments, especially nature, helps to restore attention after depletion because natural environments gently capture externally-focused (exogenous) attention processes, thereby allowing internally-focused (endogenous) attention to rest and be replenished (Kaplan and Kaplan, 1989; Kaplan, 1995; Williams et al., 2018). Sustained attention has often (Hartig et al., 1991; Berto, 2005; Craig et al., 2015; Lee et al., 2015; Schutte et al., 2017; Amicone et al., 2019), but not always (Van den Berg et al., 2003; Nguyen et al., 2018; Cassarino et al., 2019; Hicks et al., 2020; Neilson et al., 2020), been associated with performance improvement after exposure to nature. Two recent meta-analyses revealed that the effect size of nature exposure on sustained attention performance was minimal (Ohly et al., 2016; Stevenson et al., 2018) and it was noted that the mechanisms underpinning the effects of nature exposure on sustained attention performance are not well understood (Stevenson et al., 2018). Here, two different mechanisms were examined in separately—alertness and connectedness to nature.

Alertness, being ready to respond, can be divided into three forms, phasic alertness—short-term readiness to make a response following a warning signal, tonic alertness—baseline readiness linked to circadian rhythm and wakefulness, and intrinsic alertness—voluntary readiness independent of external influences (Sturm and Willmes, 2001; Unsworth et al., 2018). Arousal, the non-specific activation of the brain during the wake state of the sleep–wake cycle, overlaps conceptually with alertness, with the term alertness used when participants are performing cognitive processes (Oken et al., 2006). Alertness can be measured physiologically through the diameter of the pupil (Unsworth and Robison, 2016; Van den Brink et al., 2016; Unsworth et al., 2018) and *via* questionnaires such as the Karolinska Sleepiness Scale (Åkerstedt and Gillberg, 1990). Pupil size is partly driven by the release of noradrenalin throughout the neocortex by the locus coeruleus in the brainstem (Joshi et al., 2016). Locus coeruleus activity closely corresponds with performance on vigilance tasks in monkeys (Aston-Jones et al., 1999) and humans (Alnæs et al., 2014), pupil diameter (Alnæs et al., 2014; Joshi et al., 2016), and participant-reported arousal (Alnæs et al., 2014; Morris et al., 2020).

Two forms of pupillary response, phasic and tonic, can be measured during a task. Phasic responses reflect momentary or task-induced changes in pupil size and are considered a measure of information processing load (Beatty, 1982). Tonic responses reflect a baseline pupillary response and are considered a measure of the internal state of alertness (Bradshaw, 1967; Beatty, 1982; Laeng et al., 2012). The relations between locus coeruleus tonic activity and task performance on sustained attention tasks vary along an upside-down U shape, where high and low levels of locus coeruleus tonic activity are associated with poor task performance, but intermediate levels are associated with strong task performance (Aston-Jones and Cohen, 2005; Oken et al., 2006). Accordingly, small and large tonic pupil diameter measures are associated with attentional lapses, as indicated by increased errors, slowing of responses, and increasing variability in responding (Kristjansson et al., 2009; Unsworth and Robison, 2016; Van den Brink et al., 2016; Unsworth et al., 2018). Sustained attention performance relies on a participant’s intrinsic alertness (Oken et al., 2006) and is influenced by task-level factors including the pace (Parasuraman, 1979) and dullness of the task (Robertson et al., 1997). The task-level factors can be standardized by using one dull task, such as the Sustained Attention to Response Task (SART; Robertson et al., 1997). Performance on the SART activates the brain networks involved in sustaining attention (Manly et al., 2003) and correlates with the Cognitive Failures Questionnaire (CFQ; Broadbent et al., 1982) and the Attention-Related Cognitive Errors Scale (ARCES; Carriere et al., 2008; Smilek et al., 2010).

Three studies to date have examined the associations between nature images and pupil diameter; participants viewed photographs rated either high or low in restorative value (Nordh et al., 2010; Martínez-Soto et al., 2019; Marois et al., 2021). Nordh and colleagues noted that pupil size was smaller when participants viewed park photos they rated as more restorative. They interpreted these results to suggest that the more restorative park photos might have heightened relaxation resulting in a constriction of the pupil. The other two papers, in contrast, found that pupil diameter was larger when participants viewed high compared with low restorative photos. Martínez-Soto and colleagues interpreted these results within the context of the emotional valence of the photos, suggesting that a greater emotional response associated with higher restorative ratings of the photos was associated with a larger pupil size (Martínez-Soto et al., 2019). Marois and colleagues suggested that the nature images with higher restoration potential, the high-mystery images, may have prompter greater engagement of attention with the natural images (Marois et al., 2021). In the current study pupil diameter was used as a measure of alertness during a sustained attention task to investigate if exposure to nature was associated with sustained attention performance and modulation of alertness. Stevenson et al. (2018) determined that exposure to actual rather than virtual natural environments provided a stronger and more reliable effect on measures of working memory, attentional control, and cognitive flexibility. All the studies reviewed in the meta-analysis that measured sustained attention (vigilance) had presented participants with pictures (virtual) of environments (Stevenson et al., 2018). Here, pupil diameter was measured using a lab-based Tobii eye-tracker with a high (300 Hz) sampling rate to gain an accurate measurement of pupil size. Because of the lab-based eye-tracker, a choice was made to use pictures of nature rather than exposure to actual natural environments.

Responses to natural and urban environments may be influenced by personal characteristics including identifying with, and connecting to, nature. For example, Wilkie and Stavridou (2013) showed that identifying with the country-side was associated with higher perceived restoration for natural images and lower perceived restoration for urban images, whereas preferring the city was associated with similar restoration ratings for natural and urban images (Wilkie and Stavridou, 2013). The effects on restoration outcomes appear less clear, with a follow-up study reporting similar differences for positive mood, but not for negative mood or attention (Wilkie and Clouston, 2015). Connectedness to nature is defined as an individual’s cognitive and emotional bond to the natural world (Mayer and Frantz, 2004). Recent studies have suggested a positive association between connectedness to nature, perceived restoration (Berto et al., 2018; van den Bogerd et al., 2018) and preference for higher, possibly more naturalistic vegetation in urban settings (Lee et al., 2014). It is unclear, however, whether individuals with low connectedness to nature find non-natural settings (e.g., urban environments) restorative, as proposed by Berto et al. (2018). It may be that an individual perceives an environment to be restorative when that environment has a biophilic quality that is compatible with that person’s level of connectedness to nature (Berto et al., 2018). As the restorative potential of a setting varies from person to person (Hartig et al., 1997), perhaps for people with a weak connectedness to nature being presented with a less natural environment may allow them to experience higher perceived restoration (Berto et al., 2018). Therefore, it would be interesting to examine whether an urban environment has the potential to be perceived as restorative among those with low connectedness to nature. Exploring the relation between connectedness to nature and perceived restorativeness would provide insight into whether the perceived restoration of natural and urban environments differ for individuals with varying degrees of connectedness to nature.

Although water is present in studies investigating sustained attention and exposure to nature, little consideration has been given to the differential effect that water in landscapes or waterscapes may have had on sustained attention performance or perceived attention restoration. Behavioral evidence from studies using natural stimuli containing both vegetation and water showed restorative effects on sustained attention, attentional capacity, and the attention networks (Hartig et al., 1991; Berto, 2005; Berman et al., 2008; Mayer et al., 2009; Craig et al., 2015). In contrast, Van den Berg et al. (2003) found that videos of water in natural and urban settings had no restorative benefit on sustained attention (Van den Berg et al., 2003). Only two studies have examined specifically the effects of water views on attention performance, with neither study finding water images to be more restorative than urban or greenery images (Emfield and Neider, 2014; Nguyen et al., 2018). These studies provide equivocal evidence of the restorative effects of water views on attention performance. In terms of perceived attention restoration, several researchers have found that both natural and urban scenes with water were associated with greater perceived restorative potential than those without water (Völker and Kistemann, 1982; Purcell et al., 2001; Felsten, 2009; White et al., 2010; Grassini et al., 2019). White et al. (2010) found a dose–response effect of water on perceived restorativeness, but water-only images were perceived as less restorative than mixed and greenery-only images (White et al., 2010). These findings conflict with work by Nielson and colleagues (2017), which showed no consistent pattern in the effect or proportion of water on perceived restorativeness and no evidence of difference in perceived restorativeness between water-only and greenery-only images (Nielson et al., 2018). Views of water alone may be equivalent or possibly less restorative than views containing greenery.

This paper describes two studies that attempt to understand better the roles played by alertness and connection to nature in the interplay between sustained attention performance and exposure to nature. In Study 1, the differential effects of viewing images of meadow, water, and urban environments on sustained attention performance, alertness, and perceived restorativeness were measured. Study 2 was a modified and extended replication of Study 1 with a focus on the role of connectedness to nature on sustained attention performance and perceived restorativeness.

### Study 1

Sustained attention performance is dependent upon an appropriate level of alertness (Alnæs et al., 2014). Exposure to nature may lead to an improvement in alertness from either low (Lee et al., 2015) or a high level (Ulrich et al., 1991). Lee et al. (2015) postulated that the maintenance of sustained attention control after viewing a flowery meadow roof image, compared with a degradation in performance following the viewing a concrete roof image, was due to nature gently stimulating one’s level of alertness after becoming fatigued. Study 1 aimed to examine differential effects of viewing images of a meadow, an ocean, and an urban view on sustained attention performance, alertness, and perceived restoration. The procedure of Lee et al. (2015) was used, where participants completed the SART, then viewed an image for 40s that simulated a micro-break away from concentrating on the task, and then completed the SART again. Consistent with the literature showing the attentional benefits of exposure to green spaces, including Lee et al. (2015) and Schutte et al. (2017). Hypothesis 1 was that sustained attention performance would decline least for participants allocated the meadow image, moderately for those allocated the ocean image, and most for those allocated the urban image, as measured by SART performance. Hypothesis 2 predicted that alertness, as indexed by tonic pupil diameter, would decline least for the participants viewing the meadow image, moderately for those viewing the ocean image, and most for those viewing the urban image during the SART. In line with previous research on waterscapes (Nielson et al., 2018), Hypothesis 3 was that perceived restorativeness, as measured by scores on the Perceived Restorativeness Scale (Hartig et al., 1997), would be similar for the participants viewing the meadow and ocean images, and higher for those viewing the nature images than the urban image.

### Study 1 Method

#### Participants

Seventy-nine participants were 1st year psychology students who received course credit for their participation. Participants were randomly assigned to one of three groups (meadow, ocean, or urban), using a Latin square. Toward the end of recruitment, a small number of participants were directly allocated to the groups to achieve gender balance. Six participants were excluded because they reported taking medications that might impact their thinking skills, including medications for psychosis, depression, or anxiety. A further five participants were excluded for making 30 or more omission errors on the SART as per the procedure of Lee et al. (2015), suggesting they were not attempting the task appropriately. The final sample was 68 participants (see Table 1). Ethics approval for this study was received from the University of Melbourne Psychological Sciences Human Ethics Advisory Group (ethics approval ID 1954077.1).

**Table 1:** Descriptive statistics for participants’ demographic, KSS, ARCES, and PRS scores by Group, for Study 1.

Variable	Meadow group	Ocean group	Urban group	All	Statistical test for group difference
Number of participants	23	24	21	68	
Mean age in years (SD)	21.4 (6.9)	19.3 (1.3)	21.2 (7.0)	20.6 (5.6)	*F*(2,65) = 1.03, *p* = 0.364
Sex, count male/female	9/14	8/16	5/16	22/46	*χ*^2^(2, *N* = 68) = 1.19, *p* = 0.551
Handedness, count left/right	0/23	1/23	0/21	1/67	*χ*^2^(2, *N* = 68) = 1.86, *p* = 0.394
Mean baseline KSS (SD)	4.8 (1.3)	4.6 (1.4)	4.4 (1.6)	4.6 (1.4)	Group: *F*(2,65) = 0.24, *p* = 0.79
Mean post-intervention KSS (SD)	6.0 (1.8)	5.8 (1.8)	5.8 (1.7)	5.9 (1.8)	Time: *F*(1,65) = 49.43, *p* < 0.001^***^ Group × Time: *F*(2,65) = 0.04, *p* = 0.97
Mean ARCES (SD)	2.8 (0.5)	2.9 (0.7)	2.6 (0.4)	2.8 (0.5)	*F*(2, 65) = 2.10, *p* = 0.131
Mean PRS (SD)	3.9 (0.8)	3.8 (0.9)	2.9 (1.1)^^^	3.5 (1.0)	*F*(2,65) = 8.21, *p* < 0.001, *η_p_*^2^ = 0.20^***^
Mean Being Away PRS Subscale (SD)	3.4 (1.4)	3.4 (1.4)	2.1 (1.4)^^^	3.0 (1.5)	*F*(2,65) = 6.23, *p* < 0.003, *η_p_*^2^ = 0.16^**^
Mean Fascination PRS Subscale (SD)	4.0 (1.0)	3.8 (1.2)	3.3 (1.4)	3.7 (1.2)	*F*(2,65) = 1.92, *p* = 0.156, *η_p_*^2^ = 0.06
Median Coherence PRS Subscale (IQR)	5.5 (1.0)	5.5 (1.1)	4.0 (2.3)^^^	5.3 (1.8)	*H*(2) = 15.51, *p* < 0.001, *η_p_* = 0.21^***^
Mean Compatibility PRS Subscale (SD)	3.0 (1.0)	2.8 (1.3)	2.3 (1.3)	2.7 (1.2)	(2,65) = 2.28, *p* = 0.110, *η_p_*^2^ = 0.07

*SD, standard deviation; KSS, Karolinska Sleepiness Scale; ARCES, Attention-Related Cognitive Errors Scale; PRS, Perceived Restorativeness Scale; IQR, Interquartile Range*.

^ Urban Group score is significantly different from the Meadow and Ocean Groups. *p < 0.05, **p < 0.01, ***p < 0.001.

#### Materials and Apparatus

##### Stimuli Selection

**Figure 1:**
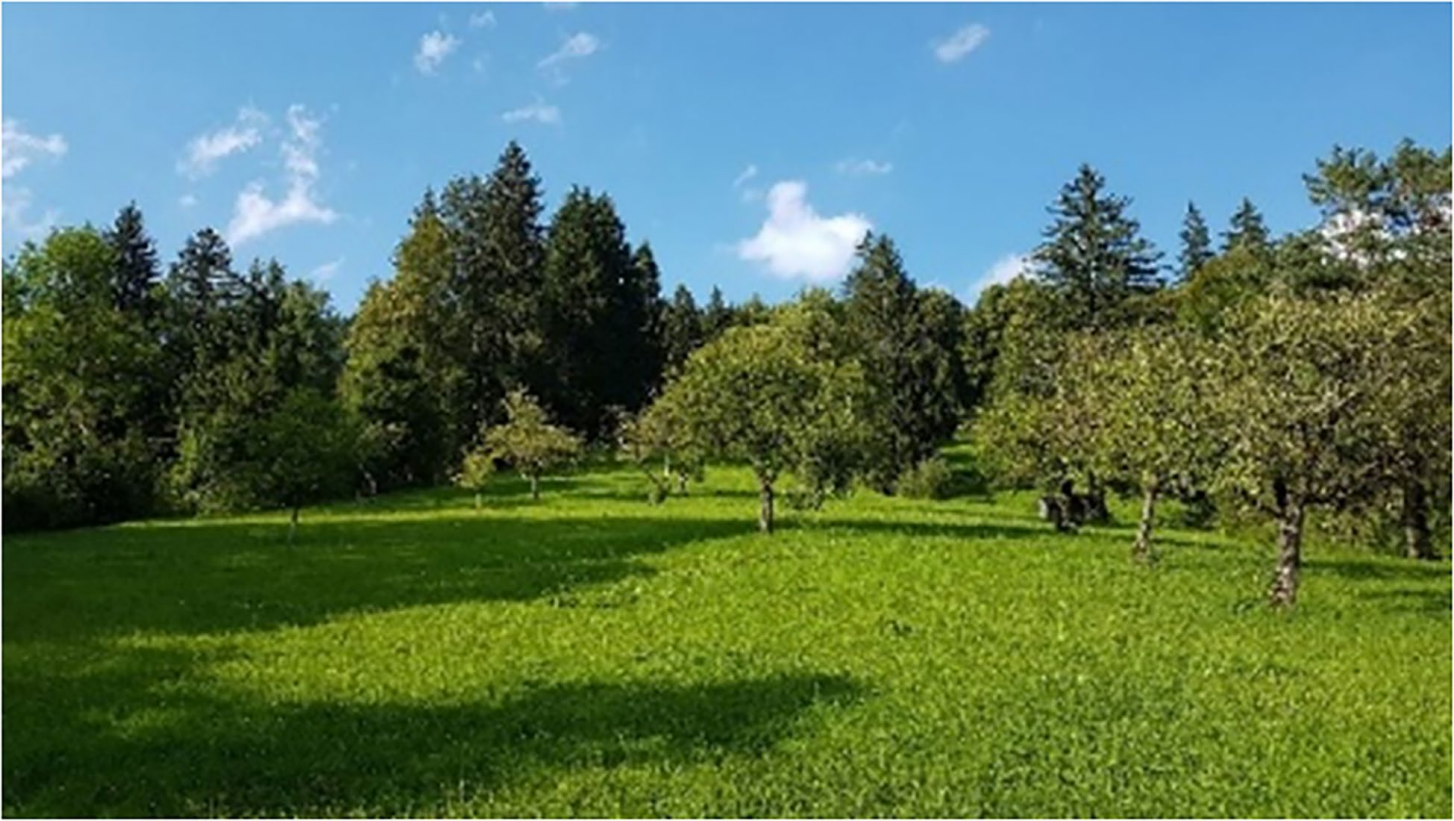
The meadow image shown for 40 s between the baseline and post-intervention SARTs in Studies 1 and 2.

**Figure 2:**
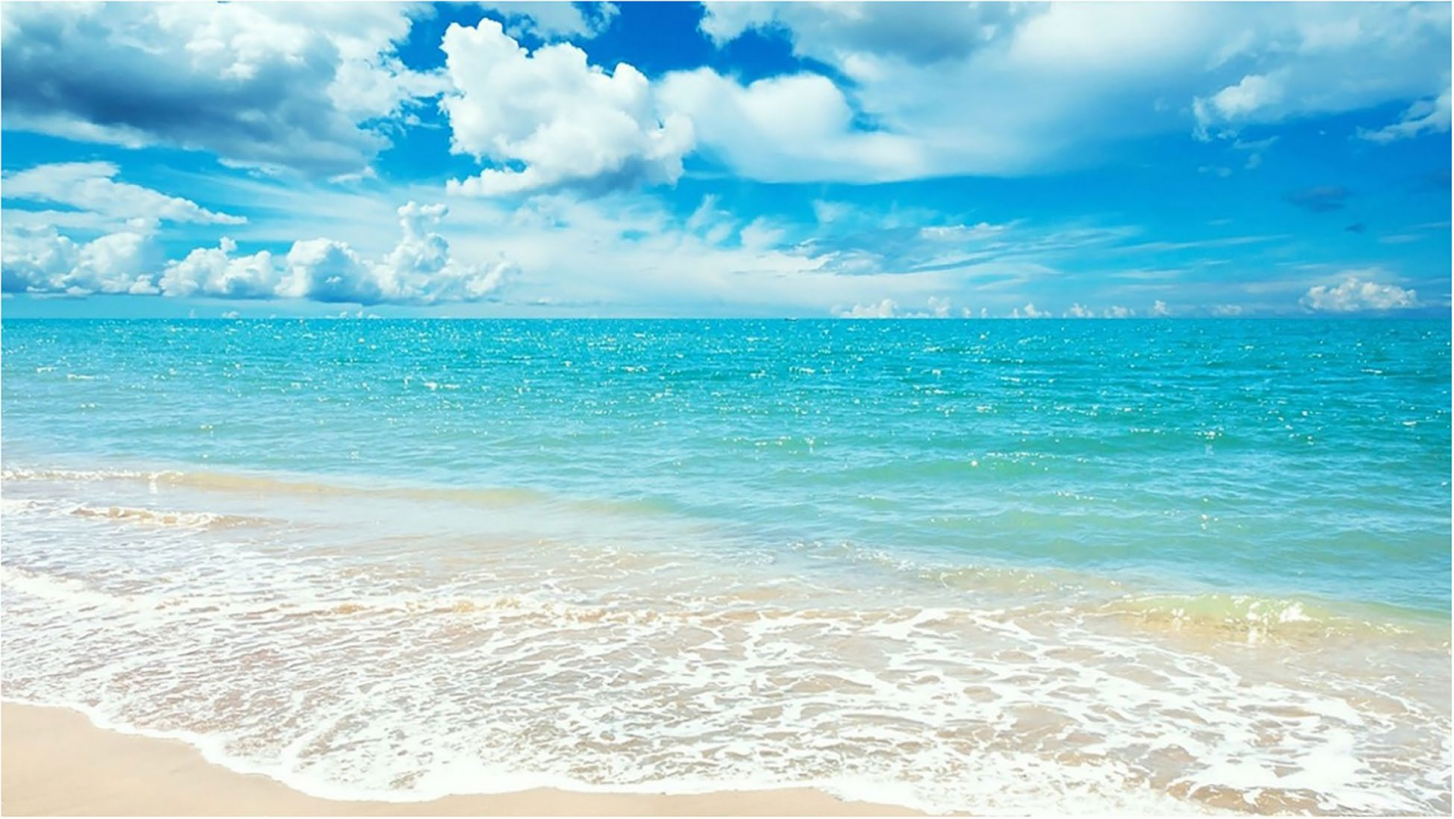
The ocean image shown for 40 s between the baseline and post-intervention SARTs in Studies 1 and 2.

**Figure 3:**
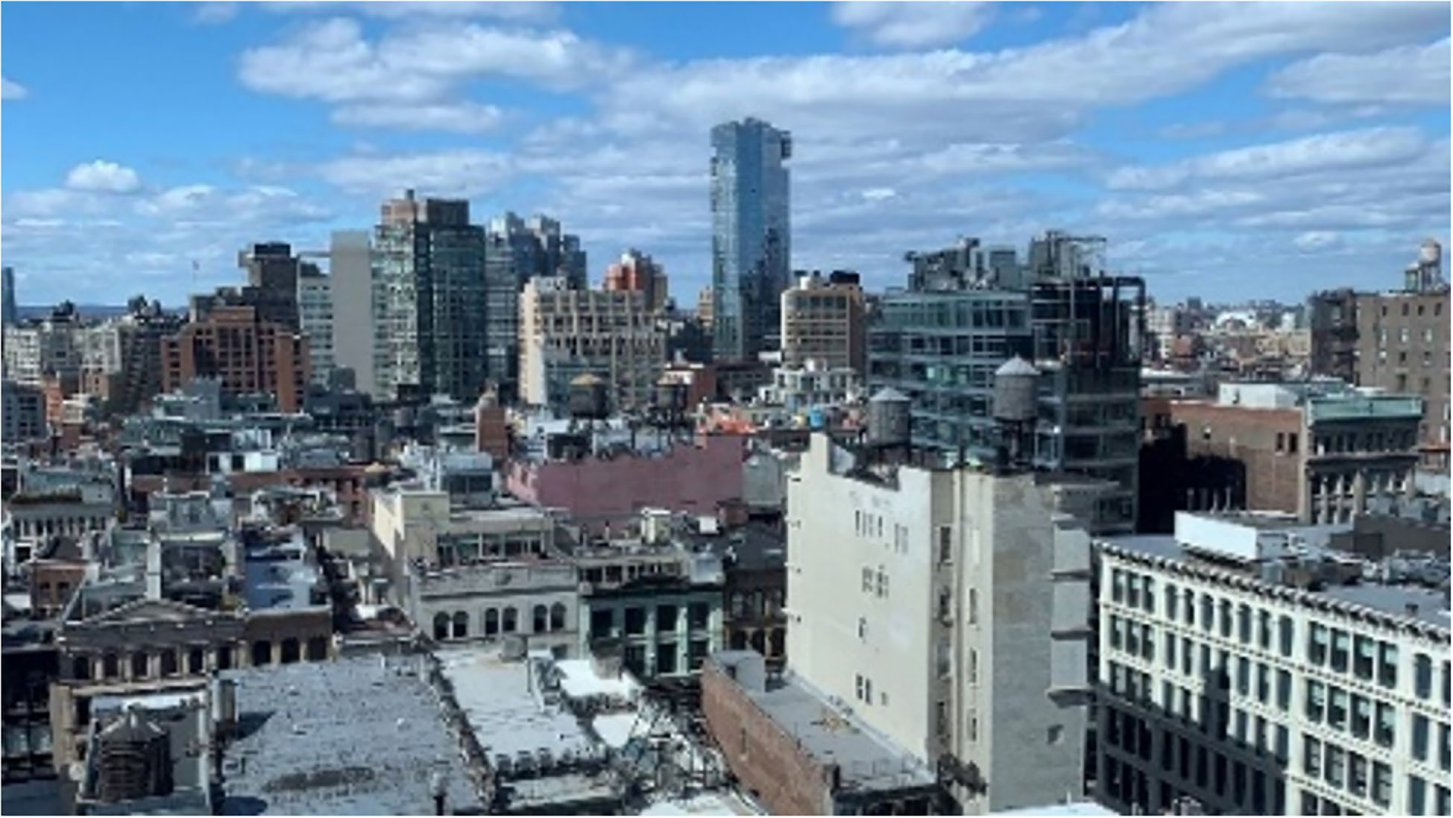
The urban image shown for 40 s between the baseline and post-intervention SARTs in Study 1.

Three images were taken from a realistic human observer perspective in similar weather conditions (see Figures 1–3). Two-thirds of each image was filled with the experimental environment (meadow, ocean, or urban) and one third with blue sky and white clouds. The three images presented similar complexity and were perceived by the authors as high in restorative characteristics (Kaplan, 1995). No people or animals were present in any of the images. Vegetation, water, and urban elements were each present in one image only and excluded from other images.

##### Scales

###### The Attention-Related Cognitive Errors Scale

The Attention-Related Cognitive Errors Scale (ARCES) is a 12-item self-report scale measuring the behavioral consequences of attention lapses in everyday situations (Carriere et al., 2008). Participants rated how frequently such attention lapses occurred to them on a 5-point Likert scale ranging from 1 (never) to 5 (very often). Scores on the revised ARCES correlate positively with errors on the SART (Cheyne et al., 2006; Smilek et al., 2010). In this dataset, the ARCES had high internal consistency, Cronbach’s *α* = 0.85.

###### The Karolinska Sleepiness Scale

The Karolinska Sleepiness Scale (KSS) is a single-item self-report scale used to assess an individual’s level of sleepiness (Åkerstedt and Gillberg, 1990). Participants rated their current level of sleepiness on a 9-point Likert scale ranging from 1 (extremely alert) to 9 (extremely sleepy).

###### The Perceived Restorativeness Scale

The Perceived Restorativeness Scale (PRS) is a 26-item measure of the perceived restorativeness of a particular environment (Hartig et al., 1997). The PRS is divided into four subscales: Being Away, Fascination, Coherence, and Compatibility. Participants used a 7-point Likert scale to rate the degree to which each of the 26 statements applied to their experience of viewing the image (0 = Not at all; 6 = Completely). In this dataset, the internal consistency of the PRS was high, Cronbach’s *α* = 0.94.

###### Sustained Attention to Response Task

The random version of the SART (Robertson et al., 1997) was administered using E-Prime 2.0 on a desktop computer with a Tobii TX300 eye-tracking monitor. Single digits from 1 to 9 appeared in the middle of the computer screen in a pseudo-random sequential order, such that the no-go “3” never appeared twice in a row. Twenty-two SART cycles of nine digits were presented, totaling 198 trials. Each trial consisted of the presentation of a fixation cross for 500 ms, followed by the digit for 150 ms, then by another fixation cross for 2,000 ms and then a variable period—a jitter, ranging between 1,170 and 1859 ms, while the fixation cross was shown. Accounting for the jitter, the inter-stimulus interval (ISI) ranged from 3,820 to 4,509 ms. The jitter was introduced to allow the pupil diameter to return to its tonic level after the visual presentation of the SART stimuli. The digits and cross were displayed in black Arial typeface on a white background. Participants completed a 9-trial practice SART, which contained one No-Go digit (“3”). The first (baseline SART) and second (post-intervention SART) SARTs each consisted of 22 No-Go trials and 176 Go trials. Each SART took approximately 14 min to complete.

###### Eye-Tracking

Pupil diameter was used as a measure of alertness. Pupil diameter was recorded using Tobii TX300, an eye-tracking device built into a 23-inch monitor. The monitor resolution was fixed at 1,920 × 1,080 pixels and the sampling rate was 300 Hz, generating 300 binocular data samples per second. A fixed chinrest, positioned 65 cm from the monitor, was used to restrict head movement and ensure optimal eye-tracking accuracy. A nine-point calibration procedure preceded each SART and image viewing.

#### Procedure

Participants were tested individually in a laboratory lit with indirect fluorescent lighting. After being informed about the study and signing a consent form, participants completed a demographic questionnaire, the KSS, and the ARCES on Qualtrics. Participants were then invited to sit in front of the computer monitor and adjust their position to fit the chinrest most comfortably. Participants received instructions to left-click on the computer mouse whenever any digit other than “3” appeared (Go trials), not to click when the digit “3” was presented (No-Go trial), and to respond as quickly and accurately as possible. Participants completed the practice SART before completing the baseline SART. After completing the baseline SART, a digital image representing a meadow, an ocean, or an urban city, was presented in full screen for 40 s, using Tobii Studio software. Participants were told that they could look freely at the image and to do nothing else during this time. After viewing the image, participants completed the post-intervention SART and then returned to Qualtrics to complete the KSS and the PRS.

#### Data Preparation

The behavioral and eye-tracking data collected *via* E-Prime and the Tobii TX300 eye-tracker were imported to MATLAB for cleaning and analysis.

##### SART Data

The SART data were prepared and analyzed according to a published method (Johnson et al., 2020), with the scripts available at DOI: 10.17605/OSF.IO/NTWY7. Data from the SARTs were divided into two (trials 0–99 and 100–198), to measure for any differences in performance across the course of the task. Time-on-task effects are an effective measure of change and potential decline in performance (Johnson et al., 2007). *Omission errors*, a failure to respond to the Go stimuli, and *commission errors*, responding to the No-Go stimulus “3,” were counted for each half of the baseline and post-intervention SARTs. After response times (RTs) less than 100 ms were removed (Luce, 1986), RTs were fitted to an ex-Gaussian distribution using maximum-likelihood-based distribution-fitting routines (Lacouture and Cousineau, 2008), and *mu*, *sigma*, and *tau* were fitted to each participant’s data set using MATLAB. Mu, sigma, and tau were calculated for each SART half. Mu and sigma represent the mean and variability of the Gaussian distribution, respectively, where tau measures the centrality of the exponential component of the RT data and represents the skewed tail of the distribution. Tau reflects very long RTs and is thought to indicate attention lapses (Leth-Steensen et al., 2000).

##### Pupil Data

Right and left pupil diameter data were analyzed separately, per participant. The data were segmented into 198 trials and analyzed on a trial-by-trial basis. Pupil data from trials with either a commission or an omission error were excluded. The data from 500 ms before stimulus onset to 2.1 s after onset were analyzed. Outliers and blinks were identified and excluded. Trials with 50% or more of the pupil data missing were noted, and individuals were excluded if more than 50% of their trials were missing. The *mean tonic pupil diameter* per trial was calculated from the 150 pupil diameter data points recorded between −500 ms (trial onset) and 0 ms (stimulus onset). These data were then averaged across the left and right eyes. The grand mean tonic pupil diameter per participant per half of each SART was then calculated. These data were then used to calculate the *linear change in mean tonic pupil diameter* by taking the coefficient of the linear regression, treating mean tonic pupil size as the outcome variable and trial order as an independent variable. The linear change in tonic pupil diameter per participant per half of each SART was then calculated.

##### Missing Data

Pupil data from the post-intervention SART were missing for one participant. After missing data was replaced with the mean values of the whole sample (calculated for each trial), analyses were run with and without this participant’s dataset. The overall pattern of results did not differ therefore this dataset was included in all analyses.

#### Statistical Analyses

The dependent variables were the errors of omission and commission, mu, sigma, tau, linear change in tonic pupil diameter, and mean tonic pupil diameter for each half of the baseline and post-intervention SARTs, the KSS at baseline and post-intervention, and the PRS scores. Each dependent variable was calculated per participant and averages were calculated for each of the three groups. Statistical analyses were conducted using R version 3.5.2, RStudio version 1.2.1320, and the lme4 and stats packages (R Core Team, 2012; Bates et al., 2020). All hypotheses were tested with an alpha set at *p* < 0.05.

Prior to hypothesis testing, any demographic and characteristic differences between the three groups were investigated using a one-way between groups analysis of variance (ANOVA) for parametric data, independent-samples Kruskal-Wallis test for non-parametric data, and Pearson’s Chi-Square test for categorical data. To examine whether participants were fatigued before the image intervention, within-group differences between the two halves of the baseline SART were investigated using a repeated measures *t*-test for parametric and related-samples Wilcoxon Signed Ranked test for non-parametric data. To test if participants were feeling sleepier after completing the two SARTs, any differences between the baseline and post-intervention KSS were tested using a repeated measures two-way ANOVA with Group and Time (baseline, post-intervention) as the factors.

Linear mixed-effects models were used to test the first and second hypotheses. Each of the SART variables (commission errors, mu, sigma, tau) and pupil variables (linear change in tonic pupil diameter, mean tonic pupil diameter), were modeled separately. The fixed effects: Baseline SART variable, Age, Sex, Group, SART Half, and the interaction between Group and SART Half, were included to explain variance in participants’ post-intervention SART and pupil measures. Each individual participant was added as a random effect. To test hypothesis 3, group differences (meadow, ocean, urban) on the PRS main and subscale scores were analyzed using a one-way ANOVA. An independent-samples Kruskal-Wallis test was used to examine group differences when violations of normality occurred. When the overall test was significant, a post-hoc Tukey HSD test was used alongside the one-way ANOVA, and Dunn’s test alongside the Kruskal-Wallis test, to perform multiple pairwise comparisons between the three groups to examine where the significant differences lay.

### Study 1 Results

#### Demographic Data and Participant Characteristics

The three groups did not differ in terms of age, sex, handedness, or ARCES (see Table 1).

#### Baseline SART Performance and Pupil Data

Descriptive statistics for each SART outcome measure are presented in Table 2. There were no baseline group differences in either SART half for any of the SART or pupil measures. Very few omission errors were made therefore no further analyses of this measure were undertaken. Taking each group separately and examining if there were differences between the two halves of the first SART, the linear change in tonic pupil diameter decreased in the first half and then increased in the second half of the baseline SART for the meadow group, *T* = 23.0, *z* = −3.79, *p* < 0.001, *r* = −0.56. Likewise, it decreased in the first half but then plateaued in the second half for the ocean, *T* = 3.0, *z* = −4.99, *p* < 0.001, *r* = −0.72, and urban groups, *T* = 4.0, *z* = −4.50, *p* < 0.001, *r* = −0.69. The mean tonic pupil diameter significantly decreased from the first to second half of the baseline SART for all three groups, meadow, *t*(22) = 2.85, *p* = 0.009, ocean, *t*(23) = 4.91, *p* < 0.001, urban, *t*(20) = 3.83, *p* = 0.001. For the SART variables, there were no significant half effects during the baseline SART for any group (see Supplementary Table S1).

**Table 2:** Descriptive statistics for each group for the SART and pupillometry variables for Study 1.

Variable	SART	Half	Meadow	Ocean	Urban	All	Group difference statistics
Omission Errors, median (IQR)	Baseline	First	0.0 (1.0)	0.0 (1.0)	0.0 (0.0)	0.0 (1.0)	H(2) = 3.34, *p* = 0.188
		Second	1.0 (1.0)	0.0 (1.0)	0.0 (1.0)	0.0 (1.0)	H(2) = 1.79, *p* = 0.410
	Post-intervention	First	0.0 (1.0)	0.0 (0.0)	0.0 (0.0)	0.0 (0.3)	
		Second	1.0 (2.0)	0.5 (1.0)	0.0 (1.0)	0.0 (1.0)	
Commission Errors, median (IQR)	Baseline	First	3.0 (4.5)	3.0 (3.3)	3.0 (3.0)	3.0 (3.5)	H(2) = 0.07, *p* = 0.966
		Second	3.0 (4.5)	4.0 (3.3)	3.0 (4.0)	3.0 (3.3)	H(2) = 0.78, *p* = 0.678
	Post-intervention	First	4.0 (4.5)	3.0 (3.3)	2.0 (4.0)	3.0 (5.0)	
		Second	4.0 (4.0)	4.5 (4.0)	3.0 (4.0)	4.0 (4.0)	
Mu (ms), median (IQR)	Baseline	First	313 (87)	304 (47)	322 (110)	308 (70)	H(2) = 1.42, *p* = 0.496
		Second	310 (74)	296 (59)	311 (69)	305 (71)	H(2) = 1.96, *p* = 0.375
	Post-intervention	First	284 (61)	282 (54)	277 (66)	283 (64)	
		Second	293 (76)	276 (48)	270 (71)	279 (71)	
Sigma (ms), median (IQR)	Baseline	First	32 (23)	28 (21)	39 (20)	36 (21)	H(2) = 2.75, *p* = 0.252
		Second	44 (29)	34 (17)	37 (29)	36 (25)	H(2) = 2.32, *p* = 0.313
	Post-intervention	First	30 (14)	30 (33)	29 (24)	29 (21)	
		Second	30 (28)	36 (24)	24 (16)	31 (26)	
Tau (ms), median (IQR)	Baseline	First	75 (64)	70 (46)	65 (59)	72 (50)	H(2) = 1.37, *p* = 0.505
		Second	89 (45)	67 (43)	79 (40)	76 (49)	H(2) = 2.66, *p* = 0.265
	Post-intervention	First	70 (47)	55 (48)	72 (49)	68 (54)	
		Second	73 (78)	67 (40)	76 (46)	74 (48)	
Linear Change in Tonic Pupil Diameter (mm), median (IQR)	Baseline	First	−0.002 (0.002)	−0.002 (0.002)	−0.002 (0.003)	−0.002 (0.002)	H(2) = 1.63, *p* = 0.443
		Second	0.001 (0.001)	0.000 (0.001)	0.000 (0.001)	0.000 (0.001)	H(2) = 3.14, *p* = 0.208
	Post-intervention	First	−0.001 (0.002)	−0.001 (0.002)	−0.001 (0.002)	−0.001 (0.002)	
		Second	0.000 (0.002)	0.000 (0.002)	0.000 (0.001)	0.000 (0.001)	
Mean Tonic Pupil Diameter (mm), mean (SD)	Baseline	First	2.68 (0.33)	2.83 (0.30)	2.76 (0.29)	2.76 (0.31)	F(2,65) = 1.35, *p* = 0.267
		Second	2.63 (0.31)	2.73 (0.29)	2.68 (0.28)	2.68 (0.29)	F(2,65) = 0.72, *p* = 0.493
	Post-intervention	First	2.67 (0.30)	2.75 (0.26)	2.70 (0.25)	2.71 (0.27)	
		Second	2.64 (0.30)	2.73 (0.28)	2.65 (0.28)	2.67 (0.29)	

*The post-intervention group differences were tested using the linear mixed models, reported in text. SART, Sustained Attention to Response Task; IQR, interquartile range; SD, standard deviation; ms, milliseconds; and mm, millimetres*.

#### KSS at Baseline and Post-intervention

There was no significant difference in the Karolinska Sleepiness Scale (KSS) score between the three groups (see Table 1). There was a significant time main effect, with all three groups indicating an increase in sleepiness after the post-intervention SART. There was no interaction between time and group.

#### Hypothesis 1: Sustained Attention Performance

There was no difference in sustained attention performance outcomes across the three groups. For the commission errors, mu, and tau, baseline SART performance was a significant predictor of performance in the post-intervention SART (see Table 3). For tau, more extremely slow responses in the second compared with the first half of the post-intervention SART were made by all groups (see Table 2). There were no other significant effects of Group, Age, Sex, SART Half, or Group by SART Half interaction for these measures. For sigma, none of the predictor variables explained a significant amount of variance in the post-intervention SART performance.

**Table 3:** Results from the Linear Mixed-Effects Models for Hypotheses 1 and 2 for Study 1.

Variable	*F*(df)	*F* value	*p*
**Errors of Commission**			
Baseline SART Commission Error Performance	*F* (1, 102)	52.75^***^	<0.001
Age	*F* (1, 49)	2.98	0.091
Sex	*F* (1, 49)	0.25	0.616
Group	*F* (2, 48)	0.98	0.381
SART Half	*F* (1, 51)	0.0001	0.993
Group x SART Half	*F* (2, 51)	1.24	0.297
**Mu**			
Baseline SART Mu Performance	*F* (1, 80)	108.11^***^	<0.001
Age	*F* (1, 46)	3.59	0.064
Sex	*F* (1, 46)	0.40	0.531
Group	*F* (2, 47)	0.45	0.638
SART Half	*F* (1, 49)	0.14	0.711
Group x SART Half	*F* (2, 49)	0.18	0.835
**Sigma**			
Baseline SART Sigma Performance	*F* (1, 127)	1.33	0.251
Age	*F* (1, 55)	0.61	0.439
Sex	*F* (1, 54)	0.04	0.840
Group	*F* (2, 55)	0.94	0.396
SART Half	*F* (1, 56)	0.0009	0.976
Group x SART Half	*F* (2, 58)	0.22	0.802
**Tau**			
Baseline SART Tau Performance	*F* (1, 96)	41.00^***^	<0.001
Age	*F* (1, 58)	0.002	0.963
Sex	*F* (1, 59)	0.47	0.498
Group	*F* (2, 59)	0.86	0.430
SART Half	*F* (1, 62)	4.56^*^	0.037
Group x SART Half	*F* (2, 60)	0.53	0.589
**Linear Change in Tonic Pupil Diameter**			
Baseline SART Linear Change in Pupil Diameter	*F* (1, 127)	18.32^***^	<0.001
Age	*F* (1, 127)	1.08	0.301
Sex	*F* (1, 127)	0.07	0.797
Group	*F* (2, 127)	0.84	0.433
SART Half	*F* (1, 127)	4.79^*^	0.030
Group x SART Half	*F* (2, 127)	0.67	0.514
**Mean Tonic Pupil Diameter**			
Baseline SART Mean Pupil Diameter	*F* (1, 70)	326.63^***^	<0.001
Age	*F* (1, 59)	1.24	0.270
Sex	*F* (1, 59)	1.36	0.248
Group	*F* (2, 59)	0.24	0.785
SART Half	*F* (1, 68)	4.44^*^	0.039
Group x SART Half	*F* (2, 62)	0.65	0.527

*df*, *degrees of freedom and SART Half, difference between the first and second half of the post-intervention SART*.

* *p < 0.05*;

** *p < 0.01*;

*** *p < 0.001*.

#### Hypothesis 2: Tonic Pupil Diameter

There was no difference in the tonic pupil diameter measures across the three groups. For both the pupil diameter measures, the baseline SART measurement was a significant predictor of the post-intervention SART measurement (see Table 3). All participants, irrespective of group, showed a decrease in linear change in tonic pupil diameter in the first half and a plateau in the second half of the post-intervention SART, while the mean pupil diameter decreased from the first to second half of the post-intervention SART (see Tables 2, 3). Group, Age, Sex, and the Group by SART Half interaction were not significant for either pupil diameter measure (see Table 3).

#### Hypothesis 3: Perceived Restorativeness

##### Complete Scale

There was a significant group difference for the mean PRS score (see Table 1). Post-hoc tests indicated that viewing the image of the meadow or the ocean was perceived as being more restorative than viewing the urban image, *p* = 0.001 and *p* = 0.005, respectively. There was no significant difference in perceived restorativeness between viewing the meadow and ocean images, *p* = 0.849.

##### Subscales

For both the Being Away and Coherence subscales, there were significant Group differences (see Table 1), in which participants rated the meadow, Being Away *p* = 0.008, Coherence *p* = 0.002, and ocean, Being Away *p* = 0.009, Coherence *p* = 0.002, images as more restorative than the urban image. No difference was found between the meadow and ocean images, Being Away *p* = 0.996, Coherence *p* = 0.999. For the Fascination and Compatibility subscales, there were no significant Group differences.

### Study 1 Discussion

Contrary to expectations, there were no group differences in performance on the SART or in pupillometry measures post the image intervention. During the baseline SART all three groups performed well and were able to maintain their performance across the two halves. The decline in mean tonic pupil diameter over the course of the baseline SART, however, suggested that all three groups were becoming less alert. During the post-intervention SART participants in all groups made more extremely long responses, as indicated by the tau measure, in the second half of the task. Tau is thought to index attentional lapses (Leth-Steensen et al., 2000). The measured increase in tau in the second half of the task is interpreted as evidence of an increase in attentional lapses, which may be a consequence of cognitive fatigue. This finding was supported by evidence of physiological lowering of alertness, with the tonic pupil size diminishing across the course of the task, in all three groups. A time-on-task effect indicated by a diminishment of pupil size over the course of the task is consistent with the literature (Unsworth and Robison, 2016; Van den Brink et al., 2016). All three groups indicated an increase in sleepiness on the KSS from before the baseline SART and immediately after the post-intervention SART, and there was no effect of the image viewed on the KSS. Nevertheless, participants reported feeling more restored after viewing either nature image, particularly in terms of Being Away and Coherence, with no differences noted between the meadow and ocean groups. Previous research most often uses nature images with a combination of water and vegetation, and this combination is perceived as most restorative (White et al., 2010). Here, with the meadow and ocean clearly differentiated, both were perceived as equally restorative. There were no differences between the three images in terms of the fascination and compatibility sub-scales of the PRS. The urban image was a scene from a rooftop, which might afford a more expansive and evocative outlook. This image was modified in Study 2 to test this interpretation. These findings suggest performing two SARTs results in a fatiguing effect, and that while viewing the nature images did not provide physiological or behavioral benefits, providing no support for Hypotheses 1 and 2, participants noted a sense of restoration from the meadow and ocean images, supporting Hypothesis 3.

### Study 2

Study 2 was a modified and extended replication of Study 1. First, eye-tracking was not performed because the study was conducted online due to the COVID-19 pandemic. Second, a ground-level version of the urban stimulus was used to address limitations associated with inconsistent visual perspectives across the three images. In Study 1, the meadow and ocean images were taken at ground-level, whereas the urban image was taken from a rooftop. A high-level perspective, such as the urban view used in Study 1, affords greater perceptual and conceptual coherence (Slepian et al., 2015), and therefore greater extent. This may have increased perceived restorativeness and influenced the results relating to the urban view in Study 1. Third, the Connectedness to Nature Scale (CNS) was included to investigate if one’s connectedness to nature was associated both with sustained attention performance and perceived restoration after viewing one of the three images. Beneficial effects of nature on sustained attention performance may depend on how much the participant felt connected to nature (Mayer and Frantz, 2004).

Keeping the hypotheses similar between the two studies based on the previous literature, Hypothesis 1 was that sustained attention performance would decline least for the meadow group, moderately for the ocean group, and most for the urban group, as measured by SART performance. Hypothesis 2 was that perceived restorativeness would be similar for the meadow and ocean groups, and higher in the nature groups than in the urban group. Hypothesis 3 predicted that connectedness to nature would moderate the relation between the type of image viewed and both sustained attention performance and perceived restoration. Higher CNS scores in the meadow and ocean groups but lower CNS scores in the urban group were expected to predict better SART performance and higher PRS scores.

### Study 2 Method

#### Participants

Two hundred and fifteen participants were from the 1st-year psychology participant pool (*n* = 182) and the broader population (*n* = 33). They had not participated in Study 1. Participants were randomly assigned to one of three groups (meadow, ocean, or urban). Toward the end of recruitment, a small number of participants were directly allocated to the ocean group to achieve approximately equal group sizes. Fifteen participants were excluded because they reported a diagnosis of a condition that might impact their thinking skills, such as depression, anxiety, or Attention Deficit Hyperactivity Disorder. One participant was excluded because they had taken medication that may have affected their cognitive performance, and 13 were excluded for making 30 or more omission errors on the SART. The final sample included 186 participants (see Table 4). Ethics approval for this study was received from the University of Melbourne Psychological Sciences Human Ethics Advisory Group (ethics approval ID 1954077.3).

**Table 4:** Descriptive statistics for participants’ demographics, KSS scores, ARCES scores, PRS scores, and CNS scores for each group, for Study 2.

Variable	Meadow group	Ocean group	Urban group	All	Group difference statistics
Number of participants	66	60	60	186	
Mean age in years (SD)	20.2 (5.9)	20.3 (4.6)	19.9 (3.7)	20.1 (4.8)	*F*(2, 183) = 0.11, *p* = 0.893
Sex, count male/female	24/42	14/46	16/44	54/132	*χ*^2^(2, *N* = 186) = 2.83, *p* = 0.243
Handedness, count left/right/either	5/59/2	4/56/0	4/54/2	13/169/4	*χ*^2^(4, *N* = 186) = 2.03, *p* = 0.730
Mean baseline KSS score (SD)	4.7 (1.7)	5.1 (1.5)	4.6 (1.6)	4.8 (1.6)	Group: *F*(2, 183) = 1.270, *p* = 0.283 Time: *F*(1,183) = 21.979, *p* < 0.001^***^
Mean post-intervention KSS score (SD)	5.2 (1.8)	5.5 (1.4)	5.3 (1.7)	5.3 (1.7)	Group × Time: *F*(2,183) = 0.611, *p* = 0.544
Mean ARCES score (SD)	2.9 (0.7)	2.9 (0.6)	2.8 (0.5)	2.9 (0.6)	*F*(2, 183) = 0.59, *p* = 0.556
Mean PRS score (SD)	3.8 (1.1)	4.1 (1.0)	2.8 (0.8)^^^	3.6 (1.1)	*F*(2,183) = 29.93, *p* < 0.001, *η_p_*^2^ = 0.25^***^
Median Being Away Subscale score (IQR)	3.7 (1.8)	3.8 (2.6)	1.9 (2.7)^^^	3.4 (2.8)	H(2) = 30.18, *p* < 0.001, *η_p_*^2^ = 0.15^***^
Mean Fascination Subscale score (SD)	3.5 (1.5)	3.8 (1.4)	2.3 (1.3)^^^	3.2 (1.6)	*F*(2,183) = 18.05, *p* < 0.001, *η_p_*^2^ = 0.16^***^
Median Coherence Subscale score (IQR)	5.3 (1.5)	5.5 (1.3)	4.5 (1.6)^^^	5.3 (1.8)	H(2) = 18.23, *p* < 0.001, *η_p_*^2^ = 0.09^***^
Mean Compatibility Subscale score (SD)	3.1 (1.4)	3.4 (1.3)	2.1 (1.0)^^^	2.9 (1.4)	*F*(2,183) = 17.09, *p* < 0.001, *η_p_*^2^ = 0.16^***^
Mean CNS score (SD)	3.5 (0.6)	3.5 (0.7)	3.3 (0.6)	3.4 (0.6)	*F*(2, 183) = 1.68, *p* = 0.190

*SD, standard deviation; IQR, interquartile range; KSS, Karolinska Sleepiness Scale; ARCES, Attention-Related Cognitive Errors Scale; PRS, Perceived Restorativeness Scale; Subscale, Subscales of the PRS; CNS, Connectedness to Nature Scale*.

^ Urban Group score is significantly different from the Meadow and Ocean Groups. *p < 0.05, **p < 0.01, ***p < 0.001.

#### Materials and Apparatus

##### Stimuli Selection

**Figure 4:**
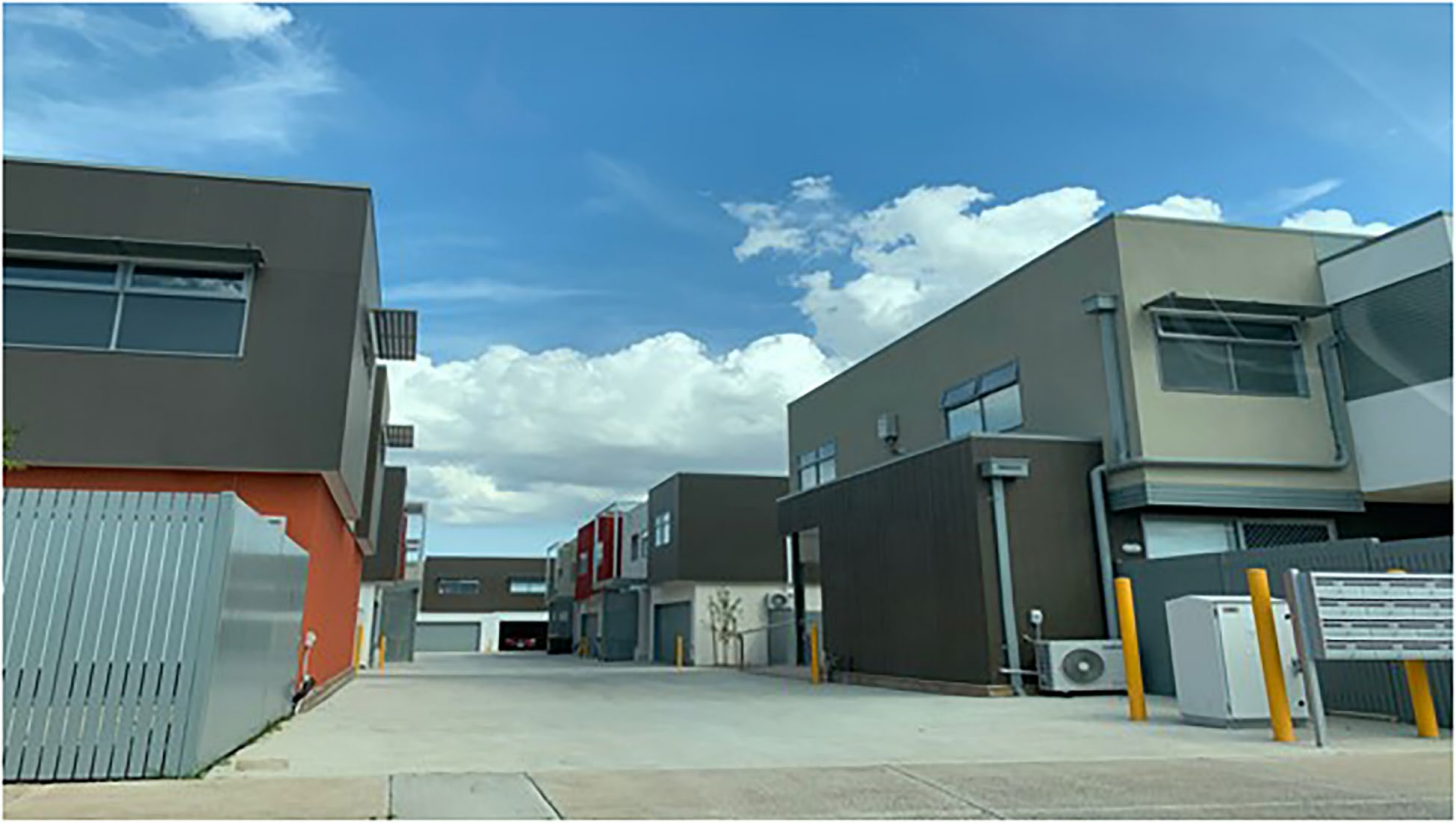
The urban image shown for 40 s between the baseline and post-intervention SARTs in Study 2.

The meadow and ocean images were taken from Study 1 and the urban image was taken from a personal collection (see Figure 4).

##### Scales

The ARCES, KSS, and PRS from Study 1 and the Connectedness to Nature Scale (CNS) were used. The CNS is a 14-item self-report scale that measures an individual’s affective connection to the natural world (Mayer and Frantz, 2004). Participants rated, on a 5-point Likert scale, the degree to which each of the 14 statements applied to them (1 = Strongly Disagree; 5 = Strongly Agree). In this dataset, the CNS had high internal consistency, Cronbach’s *α* = 0.83.

##### Sustained Attention to Response Task

The random version of the SART was administered online through Inquisit software by Millisecond. Twenty-five SART cycles of the nine digits were presented, totaling 225 trials, presented in a pseudo-randomized order. A trial sequence consisted of the digit appearing for 250 ms, followed by a mask (cross within a circle) for 900 ms. The inter-trial interval was 1,150 ms. The digits and mask were displayed in white Arial typeface on a black background. Participants completed an 18-trial practice SART, which contained two No-Go digits (“3”). The baseline and post-intervention SARTs consisted of 25 No-Go trials and 200 Go trials each. Each SART took approximately 4 min to complete.

#### Procedure

The study was administered online *via* Qualtrics and Inquisit, and participants were tested in their own time, on their own computer. All instructions were provided visually. After receiving information about the study, participants provided informed consent. Participants completed a demographic questionnaire, the KSS, and the ARCES on Qualtrics. They then installed Inquisit Player software to complete the SART. Upon starting the SART on Inquisit, participants were instructed to press the spacebar with their dominant hand’s index finger as quickly and accurately as possible every time they saw a digit other than “3” appear on the screen (Go trials) and withhold their response when the digit “3” appeared (No-Go trial). Participants completed the practice SART before completing the baseline SART. After the baseline SART, a digital image representing a meadow, an ocean, or an urban view was presented on full screen for 40 s using Inquisit. Participants were instructed to look freely at the image and to do nothing else during this time. After viewing the image, participants completed the post-intervention SART, and then returned to Qualtrics to complete the KSS, PRS, and CNS.

#### Data Preparation

The SART data were imported into MATLAB for cleaning and analysis using the same method as Study 1.

#### Statistical Analyses

The dependent variables were the errors of omission and commission, mu, sigma, and tau for each half of the baseline and post-intervention SARTs, the KSS before and after the SARTs, and the PRS scores. The moderating variable for Hypothesis 3 was the CNS score. Each dependent and moderating variable was calculated per participant and averages were calculated for each of the three groups. Statistical analyses were conducted using the same software as Study 1. All hypotheses were tested with an alpha set at *p* < 0.05.

Prior to hypothesis testing, differences between the three groups and within-group differences between the two halves of the baseline SART were investigated using the same method as Study 1. To test if participants were feeling sleepier after completing the two SARTs, any differences between the baseline and post-intervention KSS were tested using a repeated measures two-way ANOVA with group and time (baseline, post-intervention) as the factors. The same analysis techniques from Study 1 were used to test the first two hypotheses. To test Hypothesis 3, moderation analyses using multiple linear regression with an interaction term between Group and CNS scores, in predicting SART performance and PRS scores, were conducted.

### Study 2 Results

#### Demographic Data and Participant Characteristics

The three groups did not differ in terms of age, sex, handedness, mean ARCES, or mean CNS scores (see Table 4).

#### Baseline SART Performance

Descriptive statistics for each SART outcome measure are presented in Table 5. There were no baseline group differences in either SART half for any of the SART measures. Very few omission errors were made in either SART, therefore no further analyses of this measure were undertaken. Taking each group separately and examining if there were differences between the two halves of the task, sigma significantly increased for the urban group, *T* = 429, *z* = −3.58, *p* < 0.001, *r* = −0.33, and tau significantly increased for the ocean group, *T* = 625, *z* = −2.13, *p* = 0.033, *r* = −0.19. There were no other significant differences between the baseline SART halves for the other SART variables for any of the three groups (see Supplementary Table S2).

**Table 5:** Descriptive statistics of each group for the SART variables for Study 2.

Variable	SART	Half	Meadow Median (IQR)	Ocean Median (IQR)	Urban Median (IQR)	All Median (IQR)	Group difference statistics
Omission Errors (count)	Baseline	First	0.0(2.0)	0.0(2.0)	0.0(2.0)	0.0(2.0)	H(2) = 0.83, *p* = 0.659
		Second	1.0(3.0)	1.0(2.3)	0.0(2.0)	1.0(3.0)	H(2) = 3.12, *p* = 0.210
	Post-intervention	First	0.5(2.0)	0.5(2.0)	0.5(2.0)	0.5(2.0)	
		Second	1.0(4.0)	1.0(3.0)	1.0(3.0)	1.0(3.8)	
Commission Errors (count)	Baseline	First	6.0(4.8)	6.0(4.0)	7.0(4.0)	6.0(4.0)	H(2) = 0.50, *p* = 0.780
		Second	6.0(5.0)	6.0(5.0)	6.0(4.0)	6.0(5.0)	H(2) = 0.26, *p* = 0.878
	Post-intervention	First	7.0(5.0)	7.0(4.3)	7.0(5.0)	7.0(5.0)	
		Second	7.0(5.0)	7.0(5.0)	7.0(6.3)	7.0(5.0)	
Mu (ms)	Baseline	First	264(77)	270(67)	264(57)	265(62)	H(2) = 1.39, *p* = 0.498
		Second	255(100)	267(74)	269(64)	265(82)	H(2) = 0.14, *p* = 0.933
	Post-intervention	First	264(116)	258(92)	256(78)	258(93)	
		Second	274(128)	258(82)	259(105)	264(100)	
Sigma (ms)	Baseline	First	45(28)	41(29)	36(20)	41(27)	H(2) = 4.54, *p* = 0.103
		Second	49(33)	46(29)	46(22)	47(26)	H(2) = 1.21, *p* = 0.546
	Post-intervention	First	54(39)	47(28)	47(24)	47(33)	
		Second	57(47)	52(26)	56(30)	54(34)	
Tau (ms)	Baseline	First	53(48)	49(41)	56(43)	53(45)	H(2) = 2.25, *p* = 0.325
		Second	61(57)	56(51)	52(49)	56(51)	H(2) = 3.88, *p* = 0.144
	Post-intervention	First	55(58)	55(70)	52(59)	53(62)	
		Second	67(73)	71(68)	60(61)	65(68)	

*The post-intervention group differences were tested using the linear mixed models, reported in text. SART, Sustained Attention to Response Task; IQR, interquartile range; and ms, milliseconds*.

#### KSS at Baseline and Post-intervention

There was no significant difference in the Karolinska Sleepiness Scale (KSS) between the three groups. There was a significant time main effect, with all three groups indicating an increase in sleepiness after the post-intervention SART. There was no interaction between time and group.

#### Hypothesis 1: Sustained Attention Performance

There was no difference in sustained attention performance outcomes across the three groups. For all the SART measures, baseline SART performance explained variance in post-intervention SART performance (see Table 6). Younger participants made more commission errors than older participants in the post-intervention SART. Irrespective of group, participants showed a significant increase in sigma from the first to second half of the post-intervention SART. There were no other significant effects of Age or SART Half, and there were no significant effects of Group, Sex, or Group by SART Half interaction for any of the measures.

**Table 6:** Results from the Linear Mixed-Effects Models for Hypotheses 1 and 3 for Study 2.

Variable	*F*(df)	*F* value	*p*
**Errors of Commission**			
Baseline SART Commission Error Performance	*F* (1, 317)	112.88^***^	<0.001
Age	*F* (1, 142)	5.82^*^	0.017
Sex	*F* (1, 140)	0.86	0.355
Group	*F* (2, 140)	0.06	0.946
SART Half	*F* (1, 143)	0.81	0.370
Group x SART Half	*F* (2, 143)	0.72	0.489
CNS (Hypothesis 3)	*F* (1, 137)	0.03	0.853
Group x CNS (Hypothesis 3)	*F* (2, 142)	0.32	0.727
**Mu**			
Baseline SART Mu Performance	*F* (1, 335)	36.92^***^	<0.001
Age	*F* (1, 140)	3.13	0.079
Sex	*F* (1, 139)	0.18	0.668
Group	*F* (2, 139)	0.30	0.744
SART Half	*F* (1, 141)	0.34	0.562
Group x SART Half	*F* (2, 141)	0.75	0.474
CNS (Hypothesis 3)	*F* (1, 136)	1.45	0.231
Group x CNS (Hypothesis 3)	*F* (2, 136)	0.15	0.863
**Sigma**			
Baseline SART Sigma Performance	*F* (1, 360)	21.32^***^	<0.001
Age	*F* (1, 174)	0.005	0.943
Sex	*F* (1, 175)	0.06	0.809
Group	*F* (2, 175)	1.29	0.277
SART Half	*F* (1, 182)	3.93^*^	0.049
Group x SART Half	*F* (2, 176)	0.89	0.414
CNS (Hypothesis 3)	*F* (1, 171)	2.71	0.101
Group x CNS (Hypothesis 3)	*F* (2, 171)	0.862	0.444
**Tau**			
Baseline SART Tau Performance	*F* (1, 361)	38.78^***^	<0.001
Age	*F* (1, 172)	0.007	0.933
Sex	*F* (1, 172)	0.21	0.651
Group	*F* (2, 173)	0.13	0.880
SART Half	*F* (1, 176)	3.81	0.052
Group x SART Half	*F* (2, 175)	0.27	0.761
CNS (Hypothesis 3)	*F* (1, 169)	2.86	0.093
Group x CNS (Hypothesis 3)	*F* (2, 169)	0.03	0.970

*df*, *degrees of freedom and SART Half, difference between the first and second half of the post-intervention SART*.

* *p < 0.05*;

** *p < 0.01*;

*** *p < 0.001*.

#### Hypothesis 2: Perceived Restorativeness

##### Complete Scale

The mean PRS score differed significantly between the three groups (see Table 4). Post-hoc tests indicated that viewing the image of the meadow or the ocean were perceived as more restorative than viewing the urban image, both *p* < 0.001. There was no significant difference in perceived restorativeness between viewing the meadow and ocean images, *p* = 0.353.

##### Subscales

For the Being Away, Coherence, Fascination, and Compatibility subscales, there were significant Group differences, in which participants rated the meadow, Being Away *p* < 0.001, Coherence *p* = 0.005, Fascination *p* < 0.001, Compatibility *p* < 0.001, and ocean, all subscales *p* < 0.001, images as being more restorative than the urban image. No significant difference was found between the meadow and ocean images, Being Away *p* = 0.999, Coherence *p* = 0.900, Fascination *p* = 0.393, Compatibility *p* = 0.356.

#### Hypothesis 3: Connectedness to Nature, Group, and SART Performance

The CNS scores had no moderating effect on any of the SART measures. There were no significant main effects of CNS score or Group and no interactions between Group and CNS for any of the SART measures (see Table 6).

#### Hypothesis 3: Connectedness to Nature, Group, and Perceived Restorativeness

**Figure 5:**
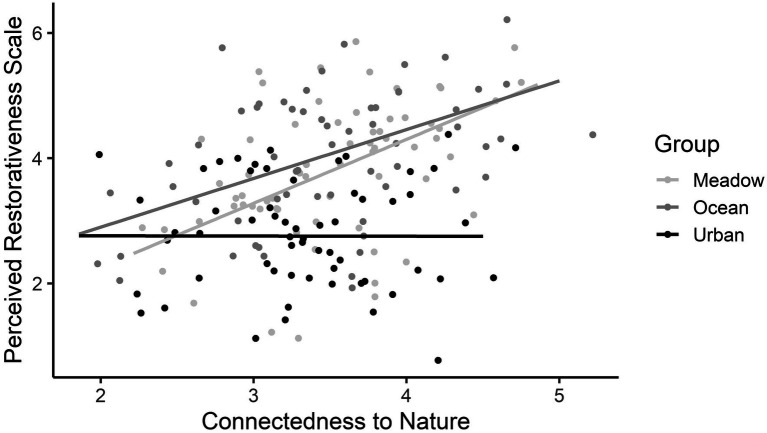
The interaction between Connectedness to Nature Scale scores and group on the Perceived Restorativeness Scale scores, from Study 2. The grey shading represents the 95% confidence intervals.

For the PRS complete scale, a main effect of Group and an interaction between Group and CNS scores were found, but there was no main effect of CNS score. For the main effect of Group, the meadow and ocean images were rated more highly on the PRS than the urban image, and the meadow and ocean images were rated similarly (see Table 7). For the interaction, participants who reported lower CNS scores were less likely to report a high PRS score after viewing their image, irrespective of their group. Those with higher reported CNS scores who viewed the meadow and ocean images rated those images with a higher PRS compared with those who viewed the urban image (see Figure 5).

**Table 7:** The moderation analysis examining the interaction between group and Connectedness to Nature Scale (CNS) scores, in predicting the Perceived Restorativeness Scale (PRS) Score in Study 2.

Predictors	Perceived Restorativeness Scale Scores					
	*b*	SE	95% CI		*t*(180)	*p*
			LL	UL		
Meadow—Urban	0.98	0.16	0.60	1.35	6.18^***^	<0.001
Ocean—Urban	1.27	0.16	0.89	1.65	7.87^***^	<0.001
Meadow—Ocean	−0.29	0.16	−0.66	0.08	−1.86	0.154
CNS Scores	−0.003	0.19	−0.38	0.38	−0.02	0.988
Meadow × CNS—Urban × CNS	1.02	0.27	0.49	1.56	3.77^***^	<0.001
Ocean × CNS—Urban × CNS	0.78	0.26	0.27	1.29	3.03^**^	0.003
Meadow × CNS—Ocean × CNS	0.24	0.26	−0.27	0.75	0.94	0.348

*b*, *beta coefficient; SE, standard error; CI, confidence intervals; LL, lower limit; UL, upper limit. Model *R*^2^ = 0.41, *F*(5, 180) = 24.87, *p* < 0.001;*

* *p < 0.05*;

** *p < 0.01*;

*** *p < 0.001*.

### Study 2 Discussion

Contrary to predictions, there were no group differences in performance on either half of the post-intervention SART. During the baseline SART there was an increase in sigma for the urban group and in tau for the ocean group, but random allocation to the three groups was used and these slight group differences occurred before presentation of the image. Like Study 1, there was no effect of the image on SART performance, providing no support for Hypothesis 1. In this faster version, with each digit appearing every 1.15 s compared with 3.8 to 4.5 s in the eye-tracking SART, all participants performed the second half of the post-intervention SART with increased sigma rather than tau, as found in Study 1. Sigma is a measure of RT variability that indexes lapses in sustained attention (Johnson et al., 2015). This change is interpreted as a sign of attentional fatigue and was noted in all three groups. As in Study 1, the baseline SART may have caused small levels of attentional fatigue that only became manifest when completing the second, post-intervention SART. Indeed, all three groups reported an increase in sleepiness from before the baseline SART to after completing the second SART, and there was no effect of the image on the KSS measure. Younger participants made more commission errors than older participants during the post-intervention SART. This observation may reflect better impulse control with age (Vallesi et al., 2021) and is consistent with research by Carriere et al. (2010) in which SART errors tended to decrease linearly with age (Carriere et al., 2010). No age effect was observed on the other SART variables.

As predicted and consistent with Study 1, the natural images were perceived as more restorative than the urban image, and the meadow and ocean images were rated as equally restorative, supporting Hypothesis 2. In contrast with Study 1, these results held true for all four subscales. This finding provides support for the speculation that the rooftop view offered by the urban image in Study 1 explained the lack of difference in fascination and compatibility between the three groups.

How connected one felt to nature was not associated with SART performance, irrespective of which image was viewed. Nevertheless, connectedness to nature did moderate the relation between viewing images of natural environments and how restorative the image was perceived. While high CNS scores predicted high PRS scores for the meadow and ocean groups, low CNS scores did not predict higher PRS scores for the urban group. The third hypothesis was partially supported. This finding is consistent with van den Bogerd et al. (2018), who found that individuals with a strong connectedness to nature rated green spaces as more restorative than individuals with low connectedness to nature (van den Bogerd et al., 2018). The finding runs against the idea that those with low connectedness to nature might have experienced higher restoration when presented with low biophilic environments, as proposed by Berto et al. (2018). Here, for participants with a weaker connectedness to nature, how restorative the image was perceived was not associated with the content of the image.

## General Discussion

Two recent meta-analyses suggested that the effects of nature exposure on sustained attention performance are small and the mechanisms underpinning this effect remain unclear (Ohly et al., 2016; Stevenson et al., 2018). The present research aimed to examine if viewing images of vegetation, water, or an urban setting led to a change in alertness (Study 1) and behavioral performance on the Sustained Attention to Response Task (SART; Studies 1 and 2), the perceived restorative value of that image (Studies 1 and 2), and the extent to which one’s connection to nature played a role in both sustained attention performance and the perceived restoration of the viewed image (Study 2). There were three key findings. First, there was no difference in the pupillometry measures of alertness, sustained attention performance, or how sleepy participants felt after exposure to the meadow, ocean, or urban environment image. Second, both natural environments were perceived as being places with more restorative potential than the urban environment, with no difference found between the meadow and ocean views. Third, stronger connectedness to nature amplified the perceived restorativeness of the nature images, but not of the urban image; this effect was not noted in the behavioral performance on the SART. This differentiation between the physiological and behavioral findings compared with the perception of the restorative value of the images is novel. The findings provide new avenues for research on the mechanisms underpinning how nature exposure may operate to restore attention.

Lee et al. (2015) speculated that the beneficial effects of viewing a flowering meadow image on sustained attention performance may have been due to gentle stimulation of alertness leading to enhanced attention control. Using an eye-tracker, the present research found that viewing nature images did not beneficially influence tonic pupil diameter, a measure of alertness. All three groups showed a decrease in linear change in tonic pupil diameter in the first half and a plateau in the second half of the post-intervention SART, and a decrease in the mean pupil diameter from the first to second half of the post-intervention SART, both of which are suggestive of a decrease in alertness. Likewise, all three groups performed the SART with increased response time variability in the second half of the post-intervention SART (tau in Study 1, sigma in Study 2), suggestive of declining sustained attention. The reason why different forms of response time variability increased over the post-intervention task between the two studies is unclear but may be due to the timing differences of the two studies. In Study 1, the inter-trial interval varied between 3.8 and 4.5 s to enable enough time between trials for the pupil size to return to the tonic, baseline, level. In contrast, Study 2 excluded pupillometry and hence, used the standard Fixed SART with an inter-trial interval of 1.15 s. The longer inter-trial interval in Study 1 may have induced more attention lapses, as indexed by more very slow responses measured using tau. The shorter inter-trial interval in Study 2 may have induced more moment-to-moment variability in responding, as indexed by sigma. Using a psychomotor vigilance task, Unsworth and colleagues reported that with a long inter-stimulus interval of 8 compared with 2 s, or with a variable interval, participants performed the task with slower responses and with smaller tonic pupil diameter, suggesting that lapses of attention are associated with lower alertness levels (Unsworth et al., 2018). In both studies all three groups reported an increase in sleepiness, measured using the KSS, following completion of the two SARTs. The pupillometry data, together with the behavioral data from the SART, and the KSS measure, suggest that participants were becoming less attentive and alert over the course of the two SARTs. The SART is a deliberately fatiguing task; a cumulative effect of performing the SART twice may have occurred. To date this is the first study to use pupillometry during a sustained attention task to test the immediate effects of viewing a nature image. The three other studies using pupillometry measured pupil diameter as participants viewed photos of different forms of nature, finding opposite effects (Nordh et al., 2010; Martínez-Soto et al., 2019; Marois et al., 2021). Here, the participants showed physiological and behavioral evidence of fatigue during both the baseline and post-intervention SARTs, and reported feeling sleepier after completing both SARTs, but with no relief proffered by viewing the nature images for the 40 s period tested here.

Regarding performance on the SART, the results from both Studies One and Two suggested that exposure to the images of nature did not influence sustained attention performance. This finding conflicts with similar research that found exposure to natural images compared with urban images led to faster response times (Berto, 2005), less response time variability and fewer omission errors (Lee et al., 2015; Cassarino et al., 2019). This finding is consistent, however, with Berto’s research in study 3 (Berto, 2005), which found no significant difference between viewing natural or urban images on any SART outcome measures, and Lee et al. (2015) who reported no difference between groups on mean response time and commission errors. It is also congruent with work by Nguyen et al. (2018), who failed to observe any restorative effect of nature images containing varying proportions of water and greenery (Nguyen et al., 2018), and Cassarino, Maisto, and colleagues (2019), who observed no differences when comparing SART performance after a simulated drive in a computerized rural or urban road environment (Cassarino et al., 2019). Together the pupil diameter and SART performance findings suggest that the mechanisms underpinning attention restoration associated with exposure to nature, using a 40 s view of a static image, are not related to alertness or cognitive functioning.

The findings from both studies strongly suggest that although the participants did not show physical or cognitive evidence of benefit after viewing the meadow or ocean images, they perceived the meadow and ocean images as places where they could feel restored. Indeed, the data from Study 2 indicated that one’s perception of restoration was tightly linked to how connected one felt to nature, as proposed by Mayer and Frantz (Mayer and Frantz, 2004). Connectedness to nature was found to moderate the relation between viewing images of natural environments and perceived restorativeness of the places in Study 2. Participants with high connectedness to nature were more likely to rate the nature images as more restorative than those with low connectedness to nature, while how connected to nature one felt was irrelevant to the restorative value of the urban image. The rooftop urban image provided some minor restorative benefits compared with the ground level urban image, with no group differences noted between the perceived restoration of the meadow, ocean, and rooftop urban images in terms of Fascination and Compatibility. The ground urban image, in contrast, did not afford such perceived benefits, with the meadow and ocean images rated as more restorative on all four subscales of the Perceived Restoration Scale. Connectedness to nature is an important moderator of perceived restorativeness of nature images. This finding highlights the important role that positive feelings and emotion play in nature-based restoration, echoing the argument put forth by colleagues that affective responses to the natural scene may explain some of the restorative value of nature (Craig et al., 2015; Martínez-Soto et al., 2019). This is an important future line of research for understanding the mechanisms associated with the Attention Restoration Theory.

In these two studies, the images presented to the participants were static photos. The Stevenson et al. (2018) meta-analysis encouraged the use of actual exposure to natural environments over the use of virtual exposure (Stevenson et al., 2018), especially in studies measuring sustained attention and vigilance. Here, a choice was made to use static images because of the use of the lab-based eye-tracker. An improvement in design in future research would include the use of mobile eye-tracking and actual exposure to different environments. Future research might also consider extending the length of duration of exposure to the different environments, as Stevenson et al. (2018) argued that longer exposure may be moderating the restorative effect of natural environments compared with control conditions. Another limitation of the two studies is that confounds associated with color, shape, shade, familiarity with the image, associations with leisure and work, heat, relaxation, and stress were not addressed. These factors could be systematically address in future research. One limitation specific to Study 2 was that the experimenters did not have control over where the participants looked when they were instructed to view the image for 40s, as the study was conducted online due to restrictions associated with COVID-19. Future research might consider broadening the concept of connection to nature to measure feelings, thoughts, and experiences people might have with nature separately, possibly through the use of the Nature Relatedness Questionnaire (Nisbet et al., 2008). These limitations and confounds may help explain why there were no physiological or behavioral differences measured between the three groups.

Here, with the use of a well-established neuropsychological test of sustained attention, alertness measured using an eye-tracker with a high sampling rate, the current best measurement of response time with the use of the Ex-Gaussian model, and a replication of the behavioral results from Study 1 in Study 2, no benefits of viewing the nature images over the urban image were measured physiologically or behaviorally. How connected one felt to nature had a strong effect on how restorative the meadow and ocean images, but not the urban image, were rated. This finding highlighted the importance of emotional response to nature, familiarity with nature, and the perspective of self within the world.

## Data Availability Statement

The datasets presented in this study can be found here: https://osf.io/56xqv.

## Ethics Statement

The studies involving human participants were reviewed and approved by University of Melbourne Psychological Sciences Human Ethics Advisory Group. The patients/participants provided their written informed consent to participate in this study.

## Author Contributions

KJ designed and directed the project. KJ, AP, and VL were involved in the planning and main conceptual ideas of the manuscript and wrote the manuscript. AP and VL collected and analyzed the data. RJ, JJ, and KM were involved in the data analysis and coding. KJ, LS, KL, NW, and KW obtained funding for the project. All authors contributed to the article and approved the submitted version.

## Funding

This paper is an output of the “Researching the benefits of demonstration green roofs across Australia (GC16002)” project funded by the Hort Frontiers Green Cities Fund, part of the Hort Frontiers strategic partnership initiative developed by Hort Innovation, with co-investment from the University of Melbourne, City of Melbourne, the Victorian Department of Environment, Land, Water and Planning and contributions from the Australian government.

## Conflict of Interest

The authors declare that the research was conducted in the absence of any commercial or financial relationships that could be construed as a potential conflict of interest.

## Publisher’s Note

All claims expressed in this article are solely those of the authors and do not necessarily represent those of their affiliated organizations, or those of the publisher, the editors and the reviewers. Any product that may be evaluated in this article, or claim that may be made by its manufacturer, is not guaranteed or endorsed by the publisher.
