# Supplementary material for: Water and Meadow Views Both Afford Perceived but Not Performance-Based Attention Restoration: Results From Two Experimental Studies
Source: Front Psychol. 2022 Apr 25;13:809629. doi: 10.3389/fpsyg.2022.809629 (PMC9084315; doi:10.3389/fpsyg.2022.809629)
Supplement: Supplementary file 1 [file Data_Sheet_1.docx]

# **Supplementary Materials**

**Supplementary Table One:** Baseline SART half effects conducted for each group separately for Study One

| Variable | Baseline SART first vs. second half  (T, z, p, r values) |
| --- | --- |
| Errors of Commission |  |
| Meadow (n = 23) | 56, -1.34, .179, -.20 |
| Ocean (n = 24) | 99, -0.23, .820, -.03 |
| Urban (n = 21) | 80, -0.63, .528, -.10 |
| Mu |  |
| Meadow (n = 23) | 149, -0.31, .754, -.05 |
| Ocean (n = 24) | 195, -1.26, .208, -.18 |
| Urban (n = 21) | 121, -0.17, .865, -.03 |
| Sigma |  |
| Meadow (n = 23) | 81, -1.72, .086, -.25 |
| Ocean (n = 24) | 146, -0.10, .922, -.01 |
| Urban (n = 21) | 144, -0.96, .338, -.15 |
| Tau |  |
| Meadow (n = 23) | 90, -1.44, .151, -.21 |
| Ocean (n = 24) | 119, -0.86, .390, -.12 |
| Urban (n = 21) | 102, -0.44, .658, -.07 |

*Note.* **p* < .05, ***p* < .01, ****p* < .001

**Supplementary Table 2:** Baseline SART half effects conducted for each group separately for Study Two.

| Variable | Baseline SART H1 vs. Baseline SART H2  (*T, z, p, r* values) |
| --- | --- |
| Errors of Commission |  |
| Meadow (*n* = 66) | 670, -1.26, .207, -.11 |
| Ocean (*n* = 60) | 689, -0.24, .812, -.02 |
| Urban (*n* = 60) | 581, -0.56, .579, -.05 |
| Mu |  |
| Meadow (*n* = 66) | 1369, -1.68, .092, -.15 |
| Ocean (*n* = 60) | 1037, -0.90, .369, -.08 |
| Urban (*n* = 60) | 746, -1.24, .214, -.11 |
| Sigma |  |
| Meadow (*n* = 66) | 857, -1.59, .112, -.14 |
| Ocean (*n* = 60) | 726, -1.39, .164, -.13 |
| Urban (*n* = 60) | 429***, -3.58, < .001, -.33 |
| Tau |  |
| Meadow (*n* = 66) | 909, -1.26, .209, -.11 |
| Ocean (*n* = 60) | 625*, -2.13, .033, -.19 |
| Urban (*n* = 60) | 960, -0.33, .740, -.03 |

*Note.* **p* < .05, ***p* < .01, ****p* < .001
